# Impact of preoperative nutritional status on colorectal surgery outcomes

**DOI:** 10.6026/973206300214289

**Published:** 2025-12-15

**Authors:** Navaneeth Ranjith, Sailesh Kumar, Shanmukha Koppolu

**Affiliations:** 1 Department of Urology, Senior Resident, Caritas Hospital, Kottayam, India; 2 Department of General Medicine, Madras Medical College, Park Town, Chennai, Tamil Nadu, India; 3 Department of Trauma and Orthopaedics, Barts NHS Trust, London, England, United Kingdom

**Keywords:** Preoperative nutrition, colorectal surgery, surgical outcomes, malnutrition, retrospective study

## Abstract

Malnutrition remains a significant yet underrecognized predictor of poor postoperative outcomes in colorectal surgery. This retrospective study analyzed the association between preoperative nutritional status and postoperative recovery in 132 patients undergoing colorectal procedures. Malnourished individuals demonstrated higher rates of surgical site infections, prolonged hospitalization and delayed wound healing compared with well-nourished patients. Nutritional screening identified 38% of patients at moderate to high risk preoperatively and mortality was notably greater in this group. These findings underscore the importance of optimizing nutritional status before surgery to improve outcomes and reduce postoperative complications.

## Background:

Colorectal surgery covers a wide range of procedures that are often linked with considerable postoperative morbidity [1]. Among the multitude of factors that influence surgical outcomes, nutritional status is a crucial, yet often overlooked, consideration [2]. Due to diminished intake, compromised absorption, or elevated metabolic burden, malnutrition is prevalent in patients with colorectal diseases, specifically malignancies, chronic inflammatory bowel disease, or obstructive lesions [3]. Preoperative malnutrition negatively impacts host immunity, diminishes muscle quantity and quality and prolongs tissue healing, which can result in an increased risk of complications, including but not limited to surgical site infections, wound complications such as dehiscence, anastomotic leaks and an increased length of stay [4]. Hypoalbuminemia, frequently used as a surrogate measure for malnutrition, has been demonstrated to have an independent relationship with increased morbidity and mortality in surgical patients [5]. Several screening instruments including Nutritional Risk Screening-2002 (NRS-2002), Subjective Global Assessment (SGA) and Malnutrition Universal Screening Tool (MUST) can be administered preoperatively [6]. However, the use of screening instruments is not uniformly adopted in surgical units [7]. Despite robust evidence linking malnutrition with poor surgical outcomes, many patients scheduled for colorectal surgery are nutritionally unoptimized at the time of the surgical intervention. Pre-emptive identification and treatment of nutritional deficiencies may improve post-surgical recovery, decrease complications and optimize overall postoperative outcomes [8]. Therefore, it is of interest to report the impact of preoperative nutritional status on postoperative outcomes in patients undergoing colorectal surgery.

## Materials and Methods:

This retrospective observational study, which involved 132 patients who had colorectal surgery between January 2020 and December 2023, was carried out in the Department of General Surgery at a tertiary care hospital. Both elective and urgent cases were taken into account. Operative notes, discharge summaries and patient medical records were the sources of the data. The Nutritional Risk Screening tool (NRS-2002) and baseline serum albumin levels were used to evaluate the nutritional status prior to surgery. Well-nourished patients (NRS ≤2 and albumin ≥3.5 g/dL) and malnourished patients (NRS >2 or albumin <3.5 g/dL) were classified. Patients who underwent neoadjuvant chemoradiation, had insufficient medical information, or had undergone reoperations for unrelated reasons were excluded [9]. Postoperative outcomes studied included surgical site infections, wound healing delays, anastomotic leakage, length of hospital stay and in-hospital mortality. Descriptive and inferential statistics were applied using SPSS version 26. Categorical variables were analyzed using the Chi-square test, while continuous variables were compared using independent t-tests or Mann-Whitney U tests as appropriate. A p-value <0.05 was considered statistically significant [10].

## Results:

Among the 132 patients undergoing colorectal surgery, 52 (39.4%) were found to be malnourished based on NRS-2002 scores and serum albumin. Compared to well-nourished patients, malnourished patients were significantly older with lower BMI, while gender and comorbidity profiles were similar between groups. Nutritional assessment revealed significantly lower albumin, hemoglobin and total lymphocyte counts in malnourished patients. Post-operative complications were higher among malnourished patients significantly for surgical site infections, delayed healing, anastomotic leaks and ICU admissions and vent support. Length of stay was greater for malnourished patients either significantly or clinically overall, with a significantly greater percentage of the sample staying >10 days. Mortality was >3x higher among malnourished patients. Reoperation rates were higher among malnourished patients, with leakage and sepsis being the leading reasons in both cohorts. Malnourished patients were also readmitted significantly more frequently within 30 days. Ultimately, this study demonstrates the importance of pre-operative nutrition screening and optimization to improve outcome measures as it relates to surgical procedures.

Table 1 (see PDF) shows that malnourished patients were significantly older with lower BMI, though gender and comorbidities did not differ notably. Table 2 (see PDF) confirms marked nutritional disparities, with malnourished patients having lower albumin, hemoglobin and lymphocyte counts alongside higher NRS-2002 scores. Postoperative outcomes were consistently poorer in this group: Table 3 (see PDF) indicates higher rates of surgical site infections across all categories and Table 4 (see PDF) shows delayed wound healing was far more common. As shown in Table 5 (see PDF), hospital stays were significantly longer in malnourished patients, while Table 6 (see PDF) highlights a greater incidence of anastomotic leaks. Table 7 (see PDF) reveals in-hospital mortality was over three times higher among malnourished patients and Table 8 (see PDF) demonstrates increased ICU admissions and ventilator support. Furthermore, Table 9 (see PDF) shows reoperation rates were higher due to complications like leaks and sepsis and Table 10 (see PDF) indicates that 30-day readmission was also significantly greater in this cohort.

## Discussion:

The results of this retrospective study underscore the significant impact of preoperative nutritional status on postoperative outcomes in patients undergoing colorectal surgery. Malnourished patients, as defined by low serum albumin and high NRS-2002 scores, experienced markedly higher rates of complications, including surgical site infections, delayed wound healing and anastomotic leaks these results are consistent with previous research that highlights malnutrition as a predictor of worse surgical outcomes that can be changed [11]. Longer hospital stays and a greater demand for ventilator use and intensive care unit support in malnourished patients further highlight the systemic vulnerability brought on by inadequate nutritional reserves Both in-hospital mortality and reoperation rates were markedly higher in the malnourished group, most likely as a result of compounded risks like sepsis and complications from leaks. The majority of patients, both malnourished and well-nourished, did not obtain proper nutritional assessment or management before surgery in spite of these hazards [12]. This points to the necessity of incorporating standardized nutritional screening instruments, such as the NRS-2002, into surgical paths and implies a serious lapse in the perioperative protocol additionally; many of the noted consequences may be lessened with early detection and focused dietary therapies, such as immunonutrition, enteral feeding, or protein-rich supplements. The need of peri-discharge nutritional planning and follow-up is further supported by the increased 30-day readmission rate in the malnourished group, which further indicates continued vulnerability after discharge. While this study is limited by its retrospective design and reliance on available records, the statistically significant differences across multiple outcome parameters support a strong correlation between nutritional status and surgical recovery. Future prospective studies with structured nutritional optimization protocols may offer more definitive evidence Nonetheless, these findings strongly advocate for making preoperative nutritional evaluation a routine part of surgical care, especially in major procedures like colorectal surgery where recovery is complex and complications are frequent [13].

## Conclusion:

Preoperative malnutrition is strongly associated with increased postoperative complications, longer hospital stays and higher mortality in colorectal surgery. Routine nutritional screening and timely intervention can significantly improve surgical outcomes. Integrating nutrition into preoperative planning should become a standard clinical practice.
